# 中国四川地区原发性非吸烟肺癌的病例对照研究

**DOI:** 10.3779/j.issn.1009-3419.2010.05.24

**Published:** 2010-05-20

**Authors:** 婷婷 蒋, 欢 宋, 夏莹 彭, 丽波 严, 敏 余, 煜 刘, 浩书 刘, 霏霏 刘, 铀 卢

**Affiliations:** 1 610041 成都，四川大学华西医院胸部肿瘤科 Department of Toracic Cancer, Cancer Center, West China Hospital, Chengdu 610041, China; 2 610041 成都，四川大学华西临床医学院 West China School of Clinical Medicine, Sichuan University, Chengdu 610041, China

**Keywords:** 肺肿瘤, 流行病学, 非吸烟, 病例对照研究, 危险因素, Lung neoplasms, Epidemiology, Non-smoking, Case-control study, Risk factors

## Abstract

**背景与目的:**

近年来非吸烟人群的肺癌发病率有上升趋势，为了给临床工作提供更准确的依据，本研究分析了四川地区原发性非吸烟肺癌的主要危险因素。

**方法:**

分别收集四川大学华西医院2009年3月-12月诊治的145例原发性非吸烟肺癌患者及145例非吸烟社区健康人群进行配对。

**结果:**

单因素分析筛选出17个相关因素；多因素条件*Logistic*回归分析显示：被动吸烟史（OR=2.267, 95%CI: 1.231-4.177）、近10年内有搬入新近装修住房史（OR=5.080, 95%CI: 1.632-15.817）、非一级血缘亲属恶性肿瘤家族史（OR=7.937, 95%CI: 1.815-34.705）、无自我解压途径（OR=2.491, 95%CI: 1.230-4.738）、工作压力大（OR=5.769, 95%CI: 2.030-16.396）、睡眠质量差（OR=2.538, 95%CI: 1.277-4.861）为主要独立危险因素；体重指数较高（OR=0.419, 95%CI: 0.226-0.779）、喜食蔬菜水果（OR=0.344, 95%CI: 0.155-0.762）、经常参加体育锻炼（OR=0.507, 95%CI: 0.274-0.937）为可能的保护因素。

**结论:**

四川地区原发性非吸烟肺癌的发生与被动吸烟史、有害环境接触史、恶性肿瘤家族史及精神心理因素等相关。

## Abstract (en)

**Background and objective:**

The incidence of lung cancer in non-smokers is increasing in recent years. The aim of this investigation is to explore main risk factors of non-smoking primary lung cancers in Sichuan province in order to provide more accurate data for clinical.

**Methods:**

One hundred and fourty-fve non-smoking pairs of cases and 145 of controls were matched by age and sex. The patients were newly-diagnosed defnitely as primary lung cancer at West China Hospital of Sichuan University from March to December 2009.

**Results:**

Seventeen exposure factors were explored as epidemic agents for non-smoking lung cancer in Sichuan by using univariate analysis; mutivariate conditional *Logistic* regression analysis showed that passive smoking, moved into newly renovated homes over the past 10 years, family cancer history from second/ third-degree relatives, lack of emotion regulation, heavy work pressure and poor quality of sleep were main risk agents for the non-smoking lung cancer incidence with OR 2.267 (95%CI: 1.231-4.177), 5.080 (95%CI: 1.632-15.817), 7.937 (95%CI: 1.815-34.705), 2.491 (95%CI: 1.230-4.738), 5.769 (95%CI: 2.030-16.396), 2.538 (95%CI: 1.277-4.861), respectively. While higher body mass index, eating fruit and vegetable and regular participating in physical exercise might be protective factors with OR 0.419 (95%CI: 0.226-0.779), 0.344 (95%CI: 0.155-0.762), 0.507 (95%CI: 0.274-0.937), respectively.

**Conclusion:**

The occurrence of non-smoking primary lung cancer associated with a variety of exposure factors including passive smoking, history of exposure to harmful environmental, family cancer history, mental and psychological factors in Sichuan Province.

肺癌是常见的恶性肿瘤之一，其高发病率及死亡率严重威胁人类生命健康。早在20世纪50年代Doll等^[1]^就提出吸烟与肺癌的发病密切相关。但并非在任何时期、任何地区、任何人群均适用这一主要病因。近年来，发展中国家非吸烟人群发生肺癌的比例也有所上升^[2]^，其潜在的发病机制也成为各国学者的研究热点。既往大量文献^[3, 4]^报道，被动吸烟是引起非吸烟人群肺癌的主要危险因素之一。而近期精神心理因素对肺癌患者的生活质量及预后的影响也逐渐引起了广泛关注^[5, 6]^。而对于四川这一特定地域有关非吸烟人群肺癌发病原因的综合性调查目前未见报道。为了寻求更准确的临床依据，本研究对四川地区非吸烟人群原发性肺癌进行问卷调查分析，旨在探讨影响该地区非吸烟人群肺癌发生的危险因素及可能的保护因素。

## 1 材料与方法

### 1.1 研究对象

本研究收集2009年3月-12月在四川大学华西第一附属医院胸部肿瘤科、呼吸科及胸外科首诊的非吸烟原发性肺癌患者151例。4例排除（3例不能排除继发性肺癌，1例为外地就诊者），2例剔除（1例无法准确理解问卷内容且家属无法提供准确信息，1例患者及家属坚决不予配合），实际纳入病例145例。纳入标准为：①组织学或细胞学确诊（仅有痰脱落细胞学病理检查不能接受。通过刷检、灌洗或穿刺获得的病理检查均可接受）的非吸烟原发性肺癌患者；②病理确诊日期在2009年1月1日以后；③无其它恶性肿瘤病史者。排除标准：①经病理检查确诊但不能排除继发性肺癌的患者；②非四川地区长期居住者。剔除标准：①患者存在肺癌脑转移或伴有精神疾病，不能正确理解问卷内容且其家属无法提供完整准确资料者；②家属及患者坚决不予配合者。同时，按性别相同、年龄±2岁、1:1配对在四川各社区选取非吸烟健康人群作为对照。对照组纳入与之匹配的非吸烟健康人群145例。具体见表 1。

**1:** 基线比较 Baseline characteristics in two groups

Characteristics	Case (*n* =145)	Control (*n* =145)
Age (year, Mean±SD)	55.56±11.791	55.67±11.670
Sex [*n* (%)]		
Male	47 (32.4)	47 (32.4)
Female	98 (67.6)	98 (67.6)

### 1.2 调查项目

此研究采用调查问卷形式进行数据采集，所用问卷由本研究小组参考国内外新近相关研究^[7-12]^统一商讨制定，包括基本资料、人口学特征、职业接触史、环境接触史、家族史、既往肺部疾病史、饮食习惯史、精神心理状态、饮酒史、规律饮用绿茶史、体育锻炼情况及女性月经生育史。

### 1.3 资料收集及审查录入

全部调查员经过统一培训，在肺癌病例入院1周内进行面访，完成有关问卷。同时按性别、年龄1:1配对的条件在四川各社区选取对照，按照相同的方式进行资料收集。2组人群问卷调查表分别由2名调查员逐一审核，并统一录入数据库。

### 1.4 统计学分析

所有数据资料均采用SPSS 13.0统计软件处理。采用*χ*^2^检验及*Monte Carlo*确切概率法、单因素条件*Logistic*回归分析法，筛选出的2组人群有统计学差异的暴露因子，再建立多因素条件Logistic回归模型，确定肺癌的可能的主要独立危险因素和保护因素。以*P* < 0.05为有统计学差异。

## 2 结果

### 2.1 单因素分析

采用*χ*^2^检验及*Monte Carlo*确切概率法、单因素条件*Logistic*回归分析，结果显示，本研究中17个暴露因素与四川地区非吸烟人群肺癌发病有关，其中7个为精神心理相关因素。本文专门针对被动吸烟史进行亚组分析，从总体人群中将鳞癌及腺癌人群按照年龄及性别分别单独进行匹配，结果提示被动吸烟史对非吸烟腺癌人群的OR值是非吸烟鳞癌人群OR值的2.37倍；男性与女性OR值较为接近，分别为3.546（*P*=0.006, 95%CI: 1.438-8.746）、2.251（*P*=0.006, 95%CI: 1.259-4.027）。具体结果见表 2及表 3。

**2:** 病例组和对照组的相关因素构成比 Cases and proportion of factors between two paired groups

Factors	Case group *n* (%)	Control group *n* (%)
Higher body mass index^$^	72 (49.7)	97 (66.9)
Passive smoking		
All cases	90 (62.1)	58 (40)
Squamous carcinoma	39 (58.2)	29 (43.3)
Adenocarcinoma	38 (66.7)	18 (31.6)
Male	23 (48.9)	10 (21.3)
Female	67 (68.4)	48 (49.0)
Lived near by (≤3 km) factories with high contamination index	32 (22.5)	15 (10.3)
Moved into newly renovated homes over the past 10 years*	28 (19.3)	5 (3.4)
Family cancer history from first-degree relatives	29 (20.0)	11 (7.6)
Family cancer history from second/third-degree relatives	10 (6.9)	3 (2.1)
History of lung disease		
All cases	41 (28.3)	23 (15.9)
COPD	5 (3.4)	7 (4.8)
Tuberculosis	14 (9.7)	6 (4.1)
Regular consumption of soy foods^**^	48 (33.1)	72 (49.7)
Eating fruit and vegetable^***^	100 (69.0)	125 (86.2)
Regular participating in physical exercise^*^	71 (49.0)	97 (66.7)
Mental and psychological factors		
Lack of emotion regulation	38 (26.2)	58 (40.0)
Characteristics		
Impatience	88 (60.7)	60 (41.4)
Irritability	49 (33.8)	31 (21.4)
Be overly concerned about trivial life	30 (20.7)	15 (10.3)
Heavy work pressure	134 (92.4)	115 (79.3)
Adequate sleep^§^	64 (44.1)	81 (55.9)
Poor quality of sleep^Ψ^	44 (30.3)	12 (8.3)
^$^Body Mass Index (BMI) > 22 kg/m^2^; The mean BMI value of case and control groups are 22.252 8±3.042 02 and 23.225 4±2.947 22 (kg/m^2^), respectively; ^§^Sleep time > 7.5 h a day. The mean sleep time of case and control groups are 7.075 9±1.651 32 and 7.524 1±1.642 88 (h), respectively; ^Ψ^Sleepless or to take medicine helping sleep more than 3 times a week; ^*^Within 3 months after the decoration; ^**^More than three times in a week; ^***^Almost eat everyday.		

**3:** *χ*^2^检验及单因素*Logistic*回归分析 Results of *Chi-square* and univariate conditional *Logistic* regression analysis

Factors	*χ*^2^	stimatied parameter *β*	Standard error	*P*	OR	95%CI
Higher body mass index^$^	8.864	-0.717	0.242	0.003	0.488	0.304-0.785
Passive smoking						
All cases	14.130	0.898	0.241	< 0.001	2.455	1.531-3.936
Squamous carcinoma^¶^	2.986	0.602	0.350	0.085	1.825	0.920-3.621
Adenocarcinoma	14.039	1.466	0.400	< 0.001	4.333	1.978-9.494
Male	7.892	1.266	0.416	0.006	3.546	1.438-8.746
Female	7.596	0.812	0.297	0.006	2.251	1.259-4.027
lived near by (≤3 km) factories with high contamination index	7.785	0.925	0.339	0.006	2.521	1.298-4.897
moved into newly renovated homes over the past 10 years^*^	18.089	1.902	0.501	< 0.001	6.701	2.508-17.903
Family cancer history from first-degree relatives	-	1.181	1.181	0.002	3.258	1.555- 6.827
Family cancer history from second/third-degree relatives	-	1.416	0.671	0.035	4.119	1.106-15.350
History of lung disease							
All cases	6.496	0.738	0.293	0.012	2.091	1.178-3.711
COPD^¶^	0.348	-0.351	0.598	0.557	0.704	0.218-2.272
Tuberculosis^¶^	3.437	0.907	0.503	0.071	2.476	0.924-6.634
Regular consumption of soy foods^**^	8.188	-0.690	0.242	0.004	0.502	0.312-0.807
Eating fruit and vegetable^***^	12.393	-1.034	0.300	0.001	0.356	0.197-0.641
Regular participating in physical exercise^*^	9.565	-0.745	0.242	0.002	0.475	0.295-0.763
Mental and psychological factors						
Lack of emotion regulation	6.229	0.630	0.254	0.013	1.877	1.142-3.087
Characteristics						
Impatience	10.818	0.783	0.239	0.001	2.187	1.368-3.497
Irritability	5.593	0.630	0.268	0.019	1.877	1.110-3.174
Be overly concerned about trivial life	5.918	0.816	0.341	0.017	2.261	1.158-4.412
Heavy work pressure	10.255	1.156	0.375	0.002	3.178	1.525-6.623
Adequate sleep^§^	3.986	0.471	0.237	0.046	0.624	0.393-0.992
Poor quality of sleep^Ψ^	22.662	1.575	0.351	< 0.001	4.828	2.425- 9.614
^$^Body Mass Index (BMI) > 22 kg/m^2^; ^§^Sleep time > 7.5 h a day; ^Ψ^Sleepless or to take medicine helping sleep more than 3 times a week; ^*^Within 3 months after the decoration; ^**^More than three times in a week; ^***^Almost eat everyday; ^¶^No statistically significant.						

### 2.2 多因素*Logistic*回归分析

在单因素分析的基础上，建立多因素条件*Logistic*回归模型，筛选出9个有统计学差异的影响四川地区肺癌发生的主要相关因素，其中体重指数较高、喜食蔬菜水果、经常参加体育锻炼为可能的保护因素。具体结果见表 4。

**4:** 多因素*Logistic*回归分析结果 Results of multivariate conditional *Logistic* regression analysis

Factors	Estimatied parameter *β*	Standard error	*P*	OR	95%CI
Higher body mass index^$^	-0.869	0.316	0.006	0.419	0.226-0.779
Passive smoking (all)	0.819	0.312	0.009	2.267	1.231-4.177
moved into newly renovated homes over the past 10 years^*^	1.625	0.579	0.005	5.080	1.632-15.817
Family cancer history from first-degree relatives^¶^	0.899	0.466	0.054	2.458	0.986-6.129
Family cancer history from second/third-degree relatives	2.071	0.753	0.006	7.937	1.815-34.705
Eating fruit and vegetable^***^	1.067	0.406	0.009	0.344	0.155-0.762
Regular participating in physical exercise	-0.680	0.314	0.030	0.507	0.274-0.937
Mental and psychological factors					
Lack of emotion regulation	0.913	0.341	0.007	2.491	1.277-4.861
Heavy work pressure	1.793	0.528	0.001	6.008	2.135-16.904
Poor quality of sleep^Ψ^	1.176	0.414	0.004	3.242	1.441-7.296
^$^Body Mass Index (BMI) > 22 kg/m^2^; ^Ψ^Sleepless or to take medicine helping sleep more than 3 times a week; ^*^Within 3 months after the decoration. ^***^ Almost eat everyday; ^¶^No statistically significant.					

## 3 讨论

据我国以往研究^[13]^结果提示，吸烟引起男性和女性肺癌的人群归因百分数（PAR%）分别为55.15%-69.10%和24.10%-40.15%，换言之，男性和女性肺癌中分别还有31.10%-44.15%及59.15%-76.10%归因于吸烟以外的其它原因。本研究共筛出17个暴露因素与四川地区非吸烟人群肺癌发病有关，其中7个为精神心理相关因素，进一步多因素分析发现9个主要相关因素，其中6个独立危险因素、3个可能的保护因素。

被动吸烟（passive smoking; second-hand smoking），又称环境烟草吸入（environmental tobacco smoke, ETS）是指生活和工作中，不自觉地吸入吸烟者吐出的烟雾尘粒和各种有毒的物质。既往文献^[14, 15]^报道其引起非吸烟人群罹患肺癌的OR值波动于1.36-3.60。在本研究中，单因素分析结果提示被动吸烟在两组非吸烟人群间存在统计学差异（*P* < 0.001），多因素*Logistic*回归分析同样也显示被动吸烟是四川地区非吸烟人群发生肺癌的独立危险因素之一（OR=2.267, 95%CI: 1.231-4.177）。近期我国卫生部报告显示，中国吸烟人数约为3.5亿，居世界各国之首，推算遭受二手烟危害的人数可高达5.4亿^[16]^，且据美国2006年卫生总监报道^[17]^：二手烟雾与吸烟者本人吸入的烟雾相比，很多致癌和有毒化学物质的浓度更高。随着我国烟草消费日益增加^[18]^，越来越多的非吸烟者可能会暴露于家庭、工作及公众的吸烟环境中，因此在提倡大家远离烟草的同时，提高公共场合的无烟意识也非常关键。本次研究针对被动吸烟史的病理类型进行了亚组单因素条件*Logistic*回归分析，结果提示被动吸烟史对非吸烟腺癌人群的OR值是非吸烟鳞癌人群的2.37倍（表 3），可见在本研究中被动吸烟更容易使非吸烟者罹患腺癌，这也与既往文献^[19, 20]^报道类似。但是就其中潜在的发病机制尚无准确结论，因此还需要更多的基础研究探索非吸烟腺癌人群的共同特征，以指导临床及公众预防工作。

有害环境接触史也是肺癌在部分特殊人群中发病率升高的原因之一^[21, 22]^。本研究多因素*Logistic*回归分析显示“近10年内有搬入新近装修住房史（装修后≤3个月入住）”为非吸烟人群罹患肺癌的独立危险因素（OR=5.080, 95%CI: 1.632-15.817; *P*=0.005）。这可能是由于长时间暴露于高浓度的甲醛、苯等来自于装修材料、家具、建材中的致癌化学物质而导致肺癌发病几率上升^[23, 24]^。

恶性肿瘤家族史与肺癌的发生发展密切相关^[4, 25]^。Gorlova等^[26]^的研究显示：非吸烟肺癌患者的一级亲属中，罹患各种癌症的风险比非吸烟健康人群的一级亲属增加25%。本研究对家族史进行了详细的分级资料采集，多因素*Logistic*回归分析结果显示一级亲属罹患恶性肿瘤家族史的非吸烟者，其罹患肺癌的OR值为2.458（95%CI: 0.986-6.129；*P*=0.054，暂无统计学意义）；而非一级血缘亲属恶性肿瘤家族史者，其OR值高达7.937（95%CI: 1.815-34.705; *P*=0.006），约为前者的3.2倍。这是否提示与肺癌发生相关的基因在隔代遗传中所起的作用更加显著？但由于目前尚未见国内外相关文献对这一特殊现象进行报道，再加之本次调查样本量较少，因此对其可能存在的原因分析还需要以后更多大规模研究的支持才能完成。

随着中国现代社会的发展，更多人群暴露于商务应酬、企业经营、人际交往、职位竞争等长期紧张的环境压力下，这使得精神心理因素逐渐成为影响人类健康的不可忽视的因素之一。同时越来越多的研究^[27]^也指出，精神心理状态不仅可能影响肺癌患者的生活质量，而且还可能进一步影响其疾病预后^[28]^。本研究针对精神心理状况方面进行了个人工作情况、睡眠情况、兴趣爱好、自我心理调节能力、性格特征、既往精神状况6个方面的调查。单因素分析筛选出7个相关因素（表 3）；进一步多因素*Logistic*回归分析结果显示（表 4）：无自我解压途径、工作压力大及睡眠质量差为肺癌的发生的独立危险因素，其OR值分别为2.491（95%CI: 1.277-4.861）、6.008（95%CI: 2.135-16.904）、3.242（95%CI: 1.441-7.296）。本文有关精神心理因素对肺癌的影响与既往国内报道^[29, 30]^类似，这可能与人体免疫系统中T细胞及NK细胞的作用相关^[31, 32]^，若及早进行心理或药物干预，可能对癌症的治疗有一定的正性作用^[33]^。

综上可见，本研究证实四川地区非吸烟人群肺癌的发生与多种暴露因素相关，在倡导公众维护室内环境及公共场所空气质量的同时，医疗工作者也应提高对患者精神心理卫生状况的关注程度，向大众普及心理卫生知识，使更多存在精神心理障碍的患者得到及时的医疗干预。
